# Association between Newborns' Breastfeeding Behaviors in the First Two Hours After Birth and Drugs Used For Their Mothers in Labor

**Published:** 2018

**Authors:** Zeinab HEMATI, Mehri ABDOLLAHI, Saba BROUMAND, Masoumeh DELARAM, Mahboobeh NAMNABATI, Davood KIANI

**Affiliations:** 1 Nursing and Midwifery Care Research Center, Isfahan University of Medical Sciences, Isfahan, Iran.; 2 Alzahra Hospital, Isfahan University of Medical Sciences, Isfahan, Iran.; 3 Department of Adult Health Nursing, Faculty of Nursing and Midwifery, Isfahan University of Medical Sciences, Isfahan, Iran.; 4 Department of Midwifery, Faculty of Nursing and Midwifery, Shahrekord University of Medical Sciences, Shahrekord, Iran.; 5 Hajar hospital, Shahrekord University of Medical Sciences, Shahrekord, Iran.

**Keywords:** Oxytocin, Hyoscine, Pethidine, Labor, Newborns' breastfeeding behaviors

## Abstract

**Objective:**

Use of narcotics to relieve pain in labor affects neurobehavioral and nutritional conditions of newborns after birth. However, there are inadequate data on the effects of drugs currently used in labor. This study was performed to examine the association between newborns' breastfeeding behaviors in the first two hours after birth and drugs used for their mothers in labor in Isfahan, central Iran, from 2014 to 2016.

**Materials & Methods:**

In this descriptive-analytical study, 300 women were selected who had vaginal delivery in the Labor and Gynecology Wards of Al-Zahra and Shahid Beheshti hospitals, Isfahan, Iran from 2014 to 2016. Data were collected by a demographic questionnaire and the Newborn Breastfeeding Behaviors Tool completed by the researcher as she observed the newborns during breastfeeding after birth. The data were analyzed by one-way ANOVA, chi-square test and Kruskal-Wallis test in SPSS 20.

**Results:**

There was no significant difference between demographic characteristics of the studied groups (*P*>0.05). In addition, there was significant difference in breastfeeding behaviors between groups. More clearly, breastfeeding ability was higher in the infants of the women administered with no drug than those of the women in the group (*P*=0.000).

**Conclusion:**

Physicians, nurses, and midwives can be informed about the side effects of the drugs used in labor on the newborns' breastfeeding, and improve their breastfeeding outcomes by decreasing the dose of used drug and the duration of the women's treatment with these drugs.

## Introduction

Breast milk is the perfect food for infants and breastfeeding rate increased from 64% in 1998 to 75% in 2010. Supply of immune system-boosting compounds, establishment of emotional relationship between the mother and infant, appropriateness of the supplied proteins and other main nutrients for the infant, supply of sufficient amounts of cholesterol and liquids in initial and final milk and most of the necessary minerals and lack of contamination are some of the benefits of breast milk (1). Decreased incidence of allergic reactions, sudden infant death syndrome, gastroenteritis, respiratory tract infection, and otitis media in the infants is another advantage of breast milk (2).

UNICEF Declaration on Breastfeeding World Week states that breastfeeding can prevent deaths of 1.3 million infants each year, and in developing countries, the infants’ breastfed are three times more likely to survive than other infants (3). To achieve these benefits, however, certain conditions such as appropriate breastfeeding ability should be realized in the infants. Women's use of drugs throughout pregnancy and in labor is one of the interventions potentially affecting the infant's breastfeeding ability. Pharmacologic interventions to relieve pain in labor, which is one of the most severe pains women experience in lifetime, can lead to reduction in the newborns' breastfeeding abilities after birth (4).

Pain relievers are one of the most important pharmacologic interventions used for women in labor. These drugs can affect the newborns' breastfeeding behaviors (5) such that they may lead to weakened breastfeeding ability of the newborns and forcing women to use other methods of feeding their infants (6). Pain relievers in labor can easily cross placenta into embryo, pass through blood-brain barrier, and affect the newborn neurobehavioral. Newborns' sleepiness immediately after delivery is one of the side effects of these drugs, which requires further attention to be paid to the doses of used drugs (7). A direct correlation between the dose of used drugs in labor and the newborns' sucking ability is reported (8).

Certain drugs such as pethidine cause decline in respiratory and muscle functions, oxygen saturation, and the newborn's heartbeat after birth. However, no definite evidence regarding weakening of the newborns' breastfeeding ability has yet been reported (7). Use of other drugs such as fentanyl to relieve pain in labor has removed sucking ability in some newborns (9). Some drugs such as hyoscine, used to relieve cervical spasms and shorten labor, can lead to fetal distress. However, further evidence is required on the effect of this drug on the newborns' breastfeeding status after birth (10). A positive correlation between use of fentanyl and decline in breastfeeding behaviors in the infants is showed (11).

Inconsistent with the current study, a study found no association between use of pain relievers in labor and the newborns' breastfeeding behaviors (12). Although the contribution of pain relievers administered to women to breastfeeding status of their infants has been reported, evidence is scant on the drugs routinely used in labor or after childbirth to relieve pain (hyoscine) and stimulate uterine contractions (oxytocin). 

Therefore, this study investigated the association between newborns' breastfeeding behaviors in the first two hours after birth and the drugs used for their mothers in labor. 

## Materials & Methods

The study population in this descriptive-analytical study was all women who had vaginal delivery in the Labor and Gynecology Wards of Al-Zahra and Shahid Beheshti hospitals, Isfahan, central Iran from 2014 to 2016. The sample size was determined as 70 in each group based on the data of similar studies: (d=. /5s^2^, α=0.05, β=0.2) 

Three hundred women were selected from this population and assigned to four groups: oxytocin + hyoscine + pethidine [70], oxytocin + pethidine [70], oxytocin + hyoscine [70] and physiologic delivery (administered with no drugs in labor) [90].

The investigated drugs are routinely used in labor and the researcher did not conduct any pharmacologic intervention. If a woman was administered with no drug, she would be assigned to the physiologic delivery group, and if she was administered with one of the above-mentioned groups of drugs, she would be assigned to the corresponding group. Mothers were selected by convenience sampling and assigned to four groups. The inclusion criteria were nulliparity, gestational age of 37-42 wk, single pregnancy, vaginal delivery, low-risk pregnancy, newborn's weight over 2500 gr, no congenital anomalies, no hospitalization in NICU, and 5-min Apgar scores ≥ 7 and the exclusion criteria exacerbated uterine contractions, mother's suffering from any medical disease and taking drug for it, taking herbal drugs to relieve pain in labor, and use of sugar water or sugar serum to feed newborn. Because the doses of the drugs used in labor and the interval between drug administration and childbirth were very important and the data would be recorded incompletely in the mothers' medical records, then two midwives who worked in the studied hospitals were asked to record the data in labor accurately. The researcher investigated the newborns' breastfeeding behaviors within two hours after birth and the mothers provided the written informed consent to participate in the study (13). 

**Ethical approval**

The researcher referred to the hospitals, explained the research purposes to the authorities of the hospitals before gathering the data and filling out the checklists after the Research and Technology Deputy of the Isfahan University of Medical Sciences provided written informed approval to conduct this study. For ethical considerations, ethical approval was issued by the Research and Technology Deputy of the Isfahan University of Medical Sciences (no.: 293258) and relevant authorities for this study.

**Data collection and analysis**

The data were gathered using a demographic questionnaire and the Newborn Breastfeeding Assessment Tool used to investigate newborns' breastfeeding in four domains: Preparing for breastfeeding, searching for nipple, sucking and putting nipple in the mouth. A four-point scale (0-3) rates the items of this questionnaire. The maximum and minimum possible score for this tool are 12 and 0, respectively. Scores ≥ 8 indicate successful breastfeeding and those <8 represent unsuccessful breastfeeding. This instrument was first used in 1988 and translated into Persian in 2009. Moreover, the validity and reliability of the Newborn Breastfeeding Assessment Tool have already been reported to be 95% for a randomly enrolled 20-individual sample (14). The data were analyzed by descriptive and analytical statistics consisting of one-way ANOVA, chi-square and Kruskal-Wallis tests by SPSS (ver. 20, Chicago, IL, USA). The level of significance was considered <0.05. For ethical considerations, ethical approval was issued by the Research and Technology Deputy of the Isfahan University of Medical Sciences (no.: 293258) and relevant authorities for this study

## Results

One-way ANOVA showed that there was no significant difference in mother’s age between four groups (*P*=0.57) (Table 1). The Chi-square test showed that there was no significant difference in the frequency of mother’s job, neonate sex and milk education between groups (*P*>0.05). In addition, the Kruskal-Wallis test showed that there was no significant difference in the mother’s education level between groups (*P*>0.05) (Table 2).

One-way ANOVA showed that there was significant difference between mean of infant's breastfeeding behaviors in the four groups (*P*<0.001). By the way, the Post hoc Tukey's test showed that the mean of infant's breastfeeding behaviors in physiologic delivery group was higher than other groups (*P*<0.001) and in oxytocin + hyoscine group was higher than two groups received pethidine (*P*=0.001) (Table 3). 

**Table 1:** Mean of mother’s age in the four groups

**Group**	**Mean ** **± SD **	***P*** **-value**
Oxytocin + hyoscine +pethidine	28.1 ± 4.6	0.57
Oxytocin +pethidine	27.9 ± 5.5	
Oxytocin + hyoscine	26.9 ± 5.8	
Physiologic delivery	27.8 ± 5.6	

**Table 2:** Frequency of demographic variables in the four groups

Variable		Oxytocin + hyoscine +pethidine N (%)	Oxytocin +pethidine N (%)	Oxytocin + hyoscine N (%)	Physiologic delivery N (%)	*P*-value
Mother’s job	Housewife	63 (90)	64 (91.4)	54 (77.1)	74 (82.2)	0.06
	Practitioner	7 (10)	6 (8.6)	16 (22.9)	16 (17.8)	
Neonate sex	Boy	33 (47.1)	34 (48.6)	33 (47.1)	38 (42.2)	0.86
	Girl	37 (52.9)	36 (51.4)	37 (52.9)	52 (57.8)	
Milk education	Yes	51 (72.9)	55 (78.6)	54 (77.1)	70 (77.8)	0.85
	No	19 (27.1)	15 (21.4)	16 (22.9)	20 (22.2)	
Mother’s education level	Illiterate	3 (4.3)	7 (10)	3 (4.3)	9 (10)	0.34
	Cycle	10 (14.3)	13 (18.6)	14 (20)	15 (16.7)	
	Diploma	36 (51.4)	35 (50)	40 (57.1)	47 (52.2)	
	Bachelor	21 (30)	13 (18.6)	11 (15.7)	18 (20)	
	Msc	0 (0)	2 (2.8)	2 (2.9)	1 (1.1)	

**Table 3:** Mean of infant's breastfeeding behaviors in the four groups

**Group**	**Mean ** **± SD **	***P*** **-value**
Oxytocin + hyoscine +pethidine	6.05 ± 2.07	<0.001
Oxytocin +pethidine	6.47 ± 2.27	
Oxytocin + hyoscine	7.78 ± 1.91	
Physiologic delivery	9.84 ± 1.64	

## Discussion

The present study was conducted to compare breastfeeding behaviours within the first two hours after birth between the newborns of the women with physiologic delivery administered with no drug and the mothers administered with drug in labor. The findings demonstrated no significant difference in demographic characteristics among the four groups (15). Moreover, the breastfeeding behaviors were significantly stronger in the infants of the mothers with physiologic delivery than those of other groups were, and the infants of the mothers administered with oxytocin + hyoscine +pethidine had the weakest breastfeeding behaviors. 

Recently, the evidence on oxytocin's role in the newborns' breastfeeding after birth has been reported to be inadequate (15). However, oxytocin use for women in labor can cause increase in lactate level in amniotic fluid, increased risk of developing certain complications such as acidosis in the newborns, delayed onset of breastfeeding, shortened duration of breastfeeding and increased feeding with milk bottle up to three months after delivery (16-20). Breastfeeding behaviors were weaker in 44% of the infants of oxytocin-administered women than the infants whose mothers were not administered with oxytocin in labor. Synthetic oxytocin seems to be able to stimulate hypoxia in the embryo to some extent through stimulating strong uterine contractions and affect the infant's breastfeeding behaviors (11). 

Moreover, increased lactate in amniotic fluid alongside academia production (21) can affect neurobehavioral status and delay onset of breastfeeding in the newborns after birth (17, 18). A review article, disconfirmed the complications following use of oxytocin use for women in labor (22).

Pethidine, as a drug used to reduce discomfort in labor, has been found to affect the newborns' breastfeeding in the first two hours after birth. Narcotics, such as pethidine, have already been found to exert adverse effects on the newborns' sucking ability and breastfeeding. A study on the effect of pethidine use on the newborns' breastfeeding behaviors demonstrated inverse correlation between mean concentration of pethidine in the infants' blood plasma and their sucking ability and breastfeeding (23).

In fact, many narcotics used in labor, such as pethidine and fentanyl, are highly lipid-soluble and result in declined neurobehavioral functions including breastfeeding via crossing the embryo's body through placenta (15, 24, 25). Decreased levels of oxytocin hormone and therefore delayed lactation in the mammary glands are some other complications due to narcotics (24). However, narcotics, including pethidine, have been reported not to exert any effects on the newborns' sucking ability after birth (26). Therefore, in the light of the present study's and other research's findings on the weakened breastfeeding of the newborns and delayed lactation due to this class of drugs, they should be used with caution, because the most important purpose after delivery is to breastfeed the newborn as immediately as possible. 

Among oxytocin, pethidine, and hyoscine, hyoscine exerted minimal side effects on the outcomes in the infants. Consistently, hyoscine has been reported to exert no effect on the embryos' heartbeat and mean Apgar score after birth (10, 27). In fact, hyoscine as a safe and the US Food and Drug Administration-approved drug to shorten the first stage of labor is compatible with the newborns' breastfeeding (10). However, the current study showed that hyoscine was effective on the infants' breastfeeding. As a result, the treatment team should be cautious in administering any drugs to women in labor in the light of hyoscine's and oxytocin's effects on the newborns' breastfeeding, because this class of drugs easily cross the embryo's body through placenta.

The most important limitation of the present study was low number of women having physiologic delivery and elongated completion of the checklist for the infants of these women. 

**In conclusion, **physicians, nurses, and midwives can be informed about the side effects of the drugs used in labor on the newborns' breastfeeding, and improve the newborns' breastfeeding outcomes by decreasing the dose of used dose and duration of women's treatment with these drugs.
